# Fat-Soluble Vitamin Supplementation Using Liposomes, Cyclodextrins, or Medium-Chain Triglycerides in Cystic Fibrosis: A Randomized Controlled Trial

**DOI:** 10.3390/nu13124554

**Published:** 2021-12-20

**Authors:** Jan K. Nowak, Paulina Sobkowiak, Sławomira Drzymała-Czyż, Patrycja Krzyżanowska-Jankowska, Ewa Sapiejka, Wojciech Skorupa, Andrzej Pogorzelski, Agata Nowicka, Irena Wojsyk-Banaszak, Szymon Kurek, Barbara Zielińska-Psuja, Aleksandra Lisowska, Jarosław Walkowiak

**Affiliations:** 1 Department of Pediatric Gastroenterology and Metabolic Diseases, Poznan University of Medical Sciences, Szpitalna 27/33, 60-572 Poznan, Poland; jan.nowak@ump.edu.pl (J.K.N.); drzymala@ump.edu.pl (S.D.-C.); pkrzyzanowska@ump.edu.pl (P.K.-J.); skurek@ump.edu.pl (S.K.); alisowska@ump.edu.pl (A.L.); 2 Department of Pneumonology, Allergology and Clinical Immunology, Poznan University of Medical Sciences, Szpitalna 27/33, 60-572 Poznan, Poland; paulinasobkowiak@ump.edu.pl (P.S.); iwojsyk@ump.edu.pl (I.W.-B.); 3 Department of Bromatology, Poznan University of Medical Sciences, Marcelinska 42, 60-354 Poznan, Poland; 4 The Specialist Centre for Medical Care of Mother and Child, Polanki 119, 80-308 Gdańsk, Poland; e.sapiejka@wp.pl; 5 Department of Lung Diseases, Institute for Tuberculosis and Lung Diseases, Plocka 26, 01-138 Warsaw, Poland; w.skorupa@igichp.edu.pl; 6 Department of Pneumology and Cystic Fibrosis, Institute of Tuberculosis and Lung Diseases, Rudnika 3, 34-700 Rabka-Zdroj, Poland; apogorzelski@igrabka.edu.pl; 7 Department of Pulmonology, Allergology and Respiratory Oncology, Poznan University of Medical Sciences, Szamarzewskiego 84, 60-569 Poznan, Poland; agnowicka@ump.edu.pl; 8 Department of Toxicology, Poznan University of Medical Sciences, Dojazd 30, 60-631 Poznan, Poland; bzielin@ump.edu.pl

**Keywords:** cystic fibrosis, vitamin A, retinol, vitamin D, cholecalciferol, vitamin E, tocopherol, vitamin K, menaquinone, liposome, cyclodextrin

## Abstract

Fat-soluble vitamin deficiency remains a challenge in cystic fibrosis (CF), chronic pancreatitis, and biliary atresia. Liposomes and cyclodextrins can enhance their bioavailability, thus this multi-center randomized placebo-controlled trial compared three-month supplementation of fat-soluble vitamins in the form of liposomes or cyclodextrins to medium-chain triglycerides (MCT) in pancreatic-insufficient CF patients. The daily doses were as follows: 2000 IU of retinyl palmitate, 4000 IU of vitamin D3, 200 IU of RRR-α-tocopherol, and 200 µg of vitamin K2 as menaquinone-7, with vitamin E given in soybean oil instead of liposomes. All participants received 4 mg of β-carotene and 1.07 mg of vitamin K1 to ensure compliance with the guidelines. The primary outcome was the change from the baseline of all-trans-retinol and 25-hydroxyvitamin D3 concentrations and the percentage of undercarboxylated osteocalcin. Out of 75 randomized patients (*n* = 28 liposomes, *n* = 22 cyclodextrins, and *n* = 25 MCT), 67 completed the trial (89%; *n* = 26 liposomes, *n* = 18 cyclodextrins, and *n* = 23 MCT) and had a median age of 22 years (IQR 19–28), body mass index of 20.6 kg/m^2^ [18.4–22.0], and forced expiratory volume in 1 s of 65% (44–84%). The liposomal formulation of vitamin A was associated with the improved evolution of serum all-trans-retinol compared to the control (median +1.7 ng/mL (IQR −44.3–86.1) vs. −38.8 ng/mL (−71.2–6.8), *p* = 0.028). Cyclodextrins enhanced the bioavailability of vitamin D3 (+9.0 ng/mL (1.0–17.0) vs. +3.0 ng/mL (−4.0–7.0), *p* = 0.012) and vitamin E (+4.34 µg/mL (0.33–6.52) vs. −0.34 µg/mL (−1.71–2.15), *p* = 0.010). Liposomes may augment the bioavailability of vitamin A and cyclodextrins may strengthen the supplementation of vitamins D3 and E relative to MCT in pancreatic-insufficient CF but further studies are required to assess liposomal vitamin E (German Clinical Trial Register number DRKS00014295, funded from EU and Norsa Pharma).

## 1. Introduction

With the advancements in cystic fibrosis (CF) care, dramatic fat-soluble vitamin deficiencies are history and current guidelines outline a mainly preventative approach to their supplementation [1]. CF patients routinely experience persistent vitamin A, D, or K insufficiency despite full compliance, raising the question of whether modern drug delivery technologies could improve the patients’ condition. For example, the use of the multivitamin A, D, E, and K tablets can augment adherence and the employment of D-α-Tocopherol polyethylene glycol 1000 succinate (TPGS) may improve vitamin E status. Formulations of both approaches have become widely available over the past decade, displacing standard mono vitamin products based on medium-chain triglycerides (MCT) that are still used for therapy individualization or cost-reduction. Despite this considerable progress, the available tools to tackle intractable vitamin deficiencies in CF remain moderate at best, hence, new delivery technologies and active compounds are urgently needed.

Liposomes are nanoscale vesicles formed by amphiphilic molecules at the border of aqueous and lipophilic compartments. They have been used to improve the pharmacokinetic properties of active compounds and reduce side effects, such as, for example, the FDA-approved intravenous doxorubicin [2]. Liposomes can be formed by soy lecithin, a common food ingredient. Cyclodextrins are glucose polymers that form hemispheric nanocontainers, with a hydrophilic exterior and an interior that can accommodate a hydrophobic compound. A range of cyclodextrins has been used in oral and intravenous drug delivery as well as in food production under the European Union (EU) Novel Foods directive [3].

Menaquinone-7 (MK-7) is a form of vitamin K naturally produced by the intestinal microflora [4] and has an almost 10-times longer serum half-life compared to phylloquinone [5]. Trials involving MK-7 have shown its safety and efficacy in osteoporosis [6,7], and it is an approved dietary supplement ingredient in the EU.

This randomized controlled trial was conducted to determine whether liposome or cyclodextrin formulations can enhance fat-soluble vitamin delivery in CF, as well as the utility of MK-7 in CF.

## 2. Materials and Methods

Pancreatic-insufficient (PI) CF patients [8,9] aged 16–55 years were eligible for the trial. The exclusion criteria were pregnancy, planned liver, or pulmonary transplant. The study was conducted in 2018 in five tertiary care centers located in four cities in Poland: the Karol Jonscher University Hospital (Poznan), the University Hospital of Lord’s Transfiguration (Poznan), the Outpatient Clinic for CF Patients (Gdansk), and the Institute of Tuberculosis and Lung Diseases (Warsaw and Rabka).

Blood was sampled following an overnight fast at inclusion and after 90 ± 4 days but not later than 1 day after the last dose of the supplement. Blood samples were immediately protected from light and centrifuged at 4–6 °C to obtain serum, which was stored at −80 °C, except during shipment on dry ice to the coordinating center (Poznan). Hemolyzed and lipemic sera were excluded.

Table 1 presents the doses of three fat-soluble vitamin supplements, namely liposomal, cyclodextrin, and MCT, that were designed and prepared for this study by Norsa Pharma (Cracov, Poland) according to the principal investigator’s (JW) recommendations. The liposomes were liquid, contained soy lecithin, and were stored in the refrigerator. Lecithin was considered free of soy allergens. Since the bulk of vitamin E proved to be too high, this vitamin was not investigated in the liposomal form; instead, patients received 200 IU of vitamin E as RRR-α-tocopherol in purified soybean oil (Tokovit E 200; Hasco-Lek, Wroclaw, Poland).

The cyclodextrin formulation was based on γ-cyclodextrin and included microcrystalline cellulose, a common food additive. It was packaged in sticks (one per vitamin, per day) and, as it was difficult to empty the sticks entirely because of the structure of the preparation, the dose packaged was increased by 10% relative to the liposomes and MCT. MCT formulations were also provided in sticks that could be emptied entirely with ease. Each patient received vitamin shipments three times in 30-day intervals to ensure adequate storage conditions and compliance with the dosing regimen. Patients ingested the vitamins during meals and were allowed to take different parts of the daily regimens at different times of the day. The vitamins were color-coded to prevent dosing errors.

Apart from the investigated vitamins, all patients also received a base daily dose of beta-carotene (4 mg; Beta-Karoten NaturKaps; Hasco-Lek, Wroclaw, Poland) and 2.5 mg of vitamin K1 (1.07 mg of phytomenadione daily; Vitacon; Polfa, Warsaw, Poland) three times a week for ethical reasons. Overall, the fat-soluble vitamin dose was concordant with the 2016 ESPEN-ESPGHAN-ECFS guidelines (Table 1). The patients were advised not to take any other vitamin preparations during the study.

All patients provided informed written consent for their participation and the study was conducted with the consent of the Bioethical Committee at Poznan University of Medical Sciences (416/17). The trial was registered in the German Clinical Trials Register (DRKS00014295), which belongs to the primary network of the World Health Organization.

The primary outcome was the mean (or median) change from the baseline in serum vitamin status. The concentrations of all-trans-retinol, α-tocopherol, and β-carotene were assessed using high-performance liquid chromatography (Hewlett Packard 1100 HPLC, Agilent, Waldbronn, Germany; Santa Clara, CA, USA). The levels of 25-hydroxyvitamin D3 were measured using chemiluminescence (Architect, Abbott, Chicago, USA). Vitamin K-dependent carboxylation was investigated using the percentage of undercarboxylated compared to total osteocalcin (%ucOC; ELISA Gla-type Osteocalcin EIA and Glu-type Osteocalcin EIA, Takara Bio Inc., Otsu, Japan).

The secondary outcomes included the change from the baseline in the prevalence of vitamin A, D, E, and K insufficiency and the analysis of the covariates on the condition that the method assumptions were met. Insufficiency was defined as <300 ng/mL of all-trans-retinol, <20 ng/mL of 25-hydroxyvitamin D3, <5 mcg/mL of alpha-tocopherol, and >20% of ucOC.

To obtain a power of 0.90 given a significance value of 0.05 and an effect size of 1 (difference of means equaling the standard deviation), the group size should be 23, with a group size of 17 allowing for comparisons with a power of 0.80. Therefore, the target recruitment was set at 75 persons, as calculated using G*Power (University of Dusseldorf, Dusseldorf, Germany).

Patients were randomly allocated to supplementation groups at a ratio of 1:1:1 using computer-generated sequences with a block size of three considering center, sex, and BMI ≥21 kg/m^2^. The patient received an individual code from their physician according to a pre-defined table that enabled the patient to register with the coordinating center via a text message or email. This triggered the dispatch of the appropriate supplement as the code was linked with the respective entry in the allocation sequence. The physician, the patient, and the laboratory staff were all blinded to group allocation, hence the study was double-blind. The MCT formulation could seem familiar to CF patients. Data and samples were collected at the study centers and the biochemical analyses were performed in the coordinating centers independent of Norsa Pharma.

If not specified otherwise, the data are presented as median (1st–3rd quartile). After checking the data for normality using the Shapiro–Wilk test, the Mann–Whitney U test was used for the primary outcome and Fisher’s exact test for the secondary outcome. Assumptions of the analysis of covariance were not met. The analyses were conducted using Statistica 13 (TIBCO Software, Palo Alto, Santa Clara, CA, USA).

## 3. Results

**Figure 1:**
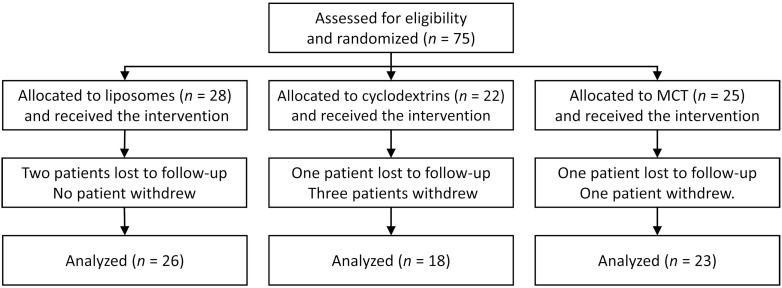
Study flowchart.

Seventy-five patients were randomized (Figure 1), of whom 67 completed the study (89%). Four patients did not return for the appointment after 90 days and four withdrew from the study (Figure 1). Among the four participants who withdrew from the study, three received the cyclodextrin formulation and one MCT. Three patients withdrew because of nausea intensifying after a few weeks of supplementation, while the fourth patient had an exacerbation and—as it was revealed later—did not take the prescribed enzymes. Although no patients withdrew from the liposomal group, two patients were lost to follow-up.

The baseline characteristics of the groups are presented in Table 2. All patients were PI and most of them were both young and had poor nutritional status. Patients in the MCT group were younger and, consequently, had a lower prevalence of *P. aeruginosa* colonization (Table 2). None of the patients received cystic fibrosis conductance regulator (CFTR) modulators or potentiators, with the homozygous F508del CFTR mutation detected in 56% of patients. The frequency of the use of the main medication groups in the overall population was 98.7% for dornase alpha, 77.3% for inhaled beta-agonists, 74.7% for ursodeoxycholic acid, 58.7% for expectorants, 44.0% for inhaled anticholinergics, 39.2% for proton-pump inhibitors, 36.0% for phospholipids, 34.7% for inhaled glucocorticoids, 29.3% for inhaled antibiotics (colistin or tobramycin), 24.0% for choline and ornithine, and 14.7% for oral antibiotics (mostly azithromycin).

**Figure 2:**
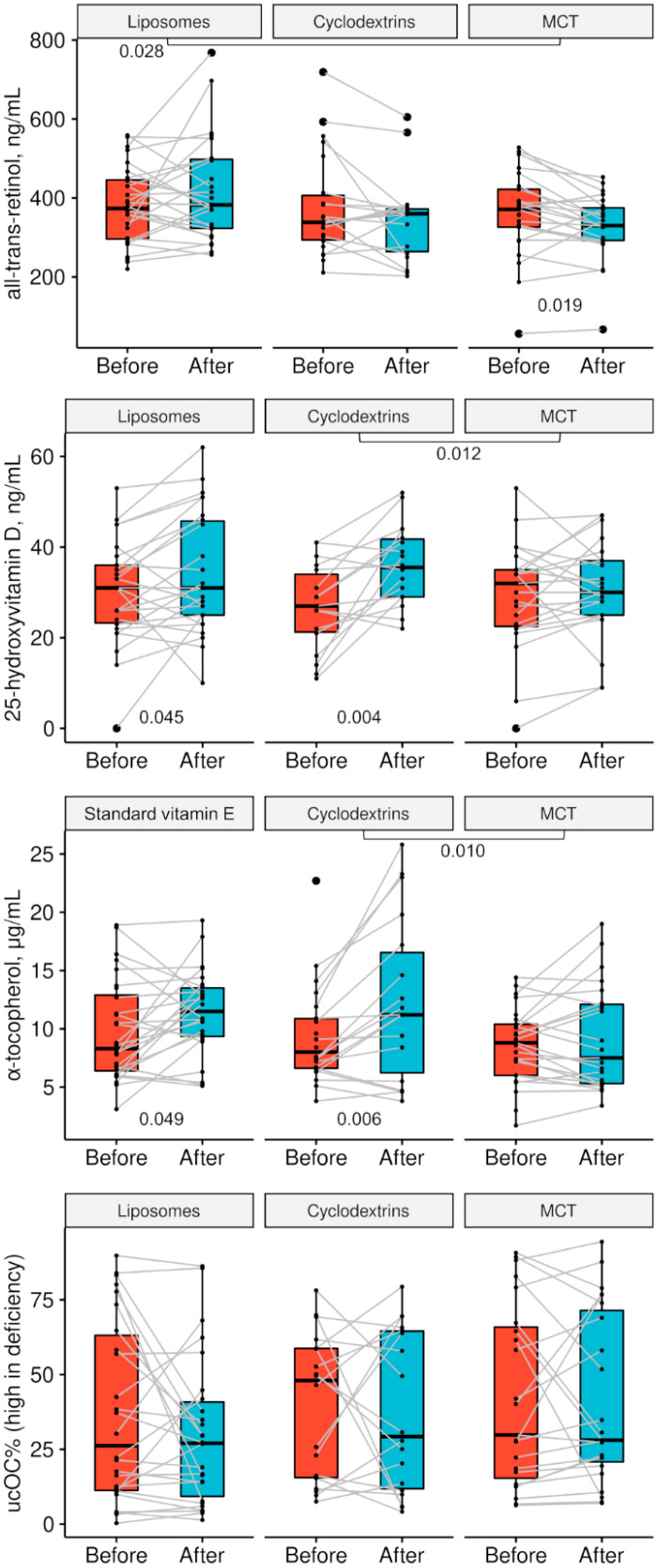
Concentrations of all-trans-retinol, 25-hydroxyvitamin D, and α-tocopherol, as well as the percentage of undercarboxylated osteocalcin (ucOC%) in PI patients with CF before and after 90 days of fat-soluble vitamin supplementation in the liposomal or cyclodextrin form and in the MCT control group. Significance of before–after differences between the groups is shown beneath group labels (Mann–Whitney U test); *p* values for comparison of vitamin levels before and after the intervention within individual groups are shown below boxplots. Boxplots illustrate the quartiles. Please note that patients in the liposome group received a standard vitamin E supplement.

Median doses of vitamin A (including both retinyl palmitate and β-carotene) before and during the study appeared similar (Table 1 and Table 3), but measurement in the serum in the MCT group indicated a higher dosage before the study (Table 4 and Figure 2). Although the median dose of vitamin D before randomization was 25% lower than that used in the study, an improvement in vitamin D3 status was not detected in the control group. The doses of vitamins E and K1 used in the study were lower than that typically used before enrollment (Table 1 and Table 3), which was reflected in the control group α-tocopherol concentrations but not %ucOC (Table 4).

**Figure 3:**
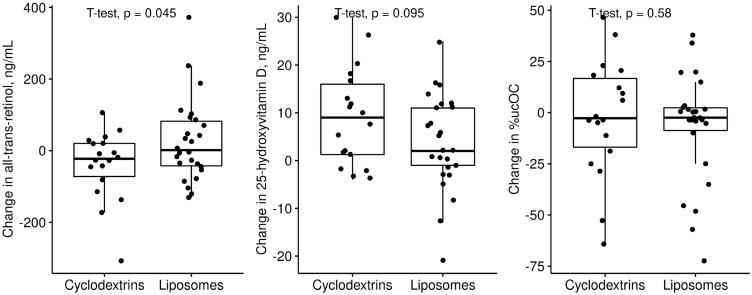
Changes in concentrations of all-trans-retinol and 25-hydroxyvitamin D, and the percentage of undercarboxylated osteocalcin (ucOC%) in PI patients with CF during the trial of the liposomal and cyclodextrin forms.

In the primary outcome analysis (Table 5 and Figure 2), supplementation of retinyl palmitate appeared more efficient compared to the control. Vitamin D3 and E in the cyclodextrin form led to higher 25-OHD3 and α-tocopherol concentrations compared to MCT. The percentage of ucOC did not differ depending on the form of menaquinone-7 or between the start and end of the intervention in all the groups. The liposomal and cyclodextrin formulations are compared with regard to the primary outcome in Figure 3.

The secondary outcome was related to changes in the frequency of vitamin insufficiencies (Table 6). In the cyclodextrin group, vitamin D insufficiency was eliminated but vitamin E insufficiency nominally increased. However, neither of these differences proved statistically significant. The percentage of vitamin K-insufficient patients was above 50% in all groups before and after the intervention.

The enhancement of vitamin D3 bioavailability in the cyclodextrin group was found to be related to the use of proton-pump inhibitors. When they were in use, no difference relative to MCT could be identified (*p* = 0.298), although the effect was strong (*p* = 0.001) in the remaining patients despite the reduced sample size.

## 4. Discussion

Fat-soluble vitamin deficiency remains a challenge for many CF patients without access to CFTR potentiators and modulators, as well as for those who exhibit insufficient vitamin levels despite such treatment [11,12,13,14]. Moreover, the problem is relevant to other diseases that involve fat maldigestion and malabsorption, such as chronic pancreatitis or biliary atresia. In this randomized study, we attempted to identify a novel formulation of fat-soluble vitamins that could prove efficient in the treatment of intractable deficiencies. To the best of our knowledge, except for TPGS, liposomal or cyclodextrin formulations of vitamins have not been explored in clinical trials. Relative to MCT, the liposomal form improved all-trans-retinol concentrations and the cyclodextrin form enhanced both serum α-tocopherol and 25-hydroxyvitamin D3.

### 4.1. Current Challenges in Fat-Soluble Supplementation

As demonstrated by historical evidence, untreated CF is associated with clinical manifestations of profound fat-soluble vitamin deficiencies. For a few decades, the key approaches to their prevention included a rich diet as well as the supplementation of pancreatic enzymes and individual vitamins [15]. To increase adherence, multivitamin preparations tailored for CF were introduced, formulated as TPGS to improve vitamin E absorption [16]. Due to potential hepatotoxicity, vitamin A levels were closely monitored and additional stress was placed on the provision of β-carotene, one of the pro-vitamins A [1]. Nevertheless, many patients continued to experience deficiencies despite supplementation, particularly with vitamin K1, which in many cases does not normalize the functional markers of protein carboxylation despite huge doses [14]. In this study, more than half of CF patients had functional vitamin K deficiency despite supplementation. We have investigated this striking phenomenon in detail and found no apparent explanation, with some effects found for apolipoprotein E polymorphisms [14,17,18,19,20].

### 4.2. New Formulations: Liposomes and Cyclodextrins

Liposome and cyclodextrin research has grown rapidly in recent years [21,22], showing their potential utility in a broad spectrum of applications from intravenous to oral and transcutaneous delivery. Liposomes encapsulate fat-soluble content and may increase delivery from an aqueous solution. Cyclodextrins are cyclic oligosaccharides forming molecular caps with a hydrophobic interior and a hydrophilic external surface. Therefore, from the perspective of fat-soluble vitamins, the promises of novel formulations include improved bioavailability and stability. A more strict dose–effect relationship might also be expected of some delivery systems, e.g., electrospun nanofibers, but more evidence is needed [23]. Increased stability was demonstrated for liposomal vitamin A [24] and cyclodextrin-complexed vitamin K3 [25], with menaquinone-4 complexed with cyclodextrins showing increased bioavailability [26]. Liposomes with beta-lactoglobulin have been investigated for vitamin A delivery [27] and liposomal vitamin E was formulated to enhance oncologic therapies [28]. The number of pharmaceutical studies of various compounds, including fat-soluble vitamins, in increasingly sophisticated novel formulations is growing rapidly but there is a lack of adequate clinical trials. Thus, the current study focused on delivery systems already approved for general use.

### 4.3. Outcomes: Vitamins A, D, E, and K

Due to the lack of data from patients who withdrew from the study, the analysis effectively took the per-protocol form. The choice of the primary endpoint (biomarker change from baseline) was dictated by the inter-patient differences in supplementation efficacy.

The improvement of vitamin A status in the liposomal form relative to MCT was considerable. To appreciate this, it is necessary to consider that larger vitamin A doses were used before compared to during the study. In the control group, all-trans-retinol concentrations decreased, whereas, despite the smaller dose, serum all-trans-retinol in the liposomal group remained constant, demonstrating the potential of the liposomal form to increase the bioavailability of retinyl palmitate. These changes could be observed despite the constant supplementation of β-carotene in all groups, which was continued for ethical reasons.

The change in the concentration of 25-hydroxyvitamin D3 was more favorable in the cyclodextrin group relative to the control. The greater efficacy of the cyclodextrin vitamin D3 in patients without proton-pump inhibitors may relate to their impact on stomach acidity or be linked to gastroesophageal reflux disease, the properties of intestinal mucus, or microbiota. Regardless of the cause, the influence of proton-pump inhibitors should be considered in future trials of oral cyclodextrin formulations.

The difference in post/pre-study vitamin E levels indicated better absorption in the cyclodextrin group. It appeared that the bioavailability of cyclodextrin vitamin E increased in patients who could already absorb it well but it was not efficacious in participants with low baseline concentrations. Vitamin E could not be included in the liposomal form because of technical difficulties. The vitamin E doses are two orders of magnitude greater than that of other vitamins, therefore they would require excessive volumes of the liposomal solution. A slight increase in the concentration of vitamin E in the group receiving a soybean oil-based formulation was found relative to MCT.

The results concerning vitamin K are especially interesting because they relate, for the first time, to the efficacy of vitamin K2 (MK-7) in CF. Apart from vitamin K1 in the standard form, all the groups received MK-7 but no improvement in functional vitamin K status was found after MK-7 supplementation. Furthermore, no differences between the three forms of MK-7 were observed (liposomes, cyclodextrins, and MCT). Some patients’ functional vitamin K status switched from low to high and inversely. Addressing vitamin K deficiency remains a challenge in CF [29], which we hope to address soon.

### 4.4. Outcomes Underscore the Need for Vitamin Dose Individualization

The control group is of interest because it demonstrates the heterogeneity of responses to fixed vitamin doses among a group of CF patients. This study provided a unique opportunity to measure how the investigated doses performed. While vitamin D and E statuses were satisfactory, vitamin A insufficiency occurred occasionally and vitamin K was often insufficient. As the doses were concordant with the current guideline [1], we hope that the presented results will inform the work on future CF nutrition guidelines.

The control group also requires attention because it was younger and had a lower prevalence of *P. aeruginosa* colonization. Such patients may be expected to absorb vitamins more easily, thus the positive influence of liposomes or cyclodextrins might be underestimated by the current study.

### 4.5. The Role of Vitamin D in CF

Vitamin D is of particular interest in CF because of its immunomodulatory and anti-inflammatory activity, as well as its significance for bone health and muscle function. Patients with CF are often vitamin D insufficient despite the use of doses higher than normally recommended for adults. This problem can be addressed by using considerably elevated doses of vitamin D3 [30]. In patients with CF-related liver disease, preparations of calcifediol are also used. Vitamin D levels in CF may modulate gut microbiota [31] and, inversely, it can also be speculated that vitamin D levels and the efficacy of its supplementation might depend on the gut microbiota. The same applies to other fat-soluble vitamins, particularly vitamin K.

### 4.6. Generalizability

The study was conducted in a group of patients receiving complex and interdisciplinary treatment, many of whom witnessed the rapid growth of capacity of the Polish healthcare system in the past two to three decades. However, none of the patients received CFTR modulators, thus this study provides information regarding the usefulness of liposomal and cyclodextrin formulations in PI CF treated with an adequate diet, pancreatic enzymes, and, in many cases, ursodeoxycholic acid. Any interaction with CFTR modulators would need to be determined even if it seems unlikely that using CFTR modulators in PI patients would significantly increase the absorption of fat-soluble vitamins. The studied population was also relatively young and with poor nutritional status in some cases. The incidence of CF-related liver disease may have been overestimated because of the high sensitivity of criteria [10]. Overall, the results are best generalizable to populations that do not yet have access to CFTR modulators but their applicability in patients receiving modulators cannot be excluded.

The knowledge of the responsiveness of individual patients to each fat-soluble vitamin might potentially enrich the interpretation of study results. However, such responsiveness is difficult to define or measure despite much research [11,13,14,18] and in clinical practice, no specific assessment of fat-soluble vitamin responsiveness is used. Instead, the doses are adjusted in each CF patient until satisfactory results are obtained and thus reflect individual needs of the patient. In this study, the doses of fat-soluble vitamins taken by patients prior to the study were very similar (*p* close to unity) and it is therefore highly unlikely that any individual responsiveness would present an important source of confounding.

The study had several advantages: (a) investigation of three (liposomal) and four (cyclodextrin) fat-soluble vitamins in two novel formulations; (b) a randomized controlled design with stratification; (c) comparing changes in vitamin status biomarkers between groups, which minimizes the impact of interindividual variability; and (d) additional inclusion of vitamin K2 as MK-7 for before–after analyses.

## 5. Conclusions

The liposomal formulation may augment the bioavailability of vitamin A and cyclodextrin formulation may strengthen the supplementation of vitamins D3 and E relative to MCT in PI patients with CF.

## Figures and Tables

**Table 1:** Daily vitamin doses in the study groups.

Vitamin	Liposomes	Cyclodextrins	MCT
A, retinyl palmitate	2000 IU	2000 IU	2000 IU
Vitamin D3	4000 IU	4000 IU	4000 IU
E, RRR-α-tocopherol	-	200 IU	200 IU
K2, menaquinone-7	200 µg	200 µg	200 µg
Vitamins in standard form			
A, β-carotene (4 mg = 6667 IU)	4 mg	4 mg	4 mg
E, RRR-α-tocopherol	200 IU	-	-
K1, phylloquinone (2.5 mg thrice per week)	1.07 mg	1.07 mg	1.07 mg

MCT—medium-chain triglycerides.

**Table 2:** Group characteristics at baseline. Medians (1st–3rd quartiles) are shown along with the *p*-value for the Kruskal–Wallis test or the Pearson’s χ2 test.

Parameter	Liposomes (*n* = 28)	Cyclodextrins (*n* = 22)	MCT (*n* = 25)	*p*
Age, years	23.1 (21.2–30.2)	23.2 (18.7–28.8)	20.4 (17.5–22.9)	0.035
Sex: female	53.6%	63.6%	72.0%	0.381
Mass, kg	58.5 (52.0–64.0)	54 (48.5–60.0)	57.0 (48.0–65.0)	0.340
Height, cm	167 (161–175.5)	165 (158–173)	168 (160–173)	0.551
BMI, kg/m^2^	20.9 (19.5–22.4)	20.4 (18.3–21.5)	20.5 (18.4–21.7)	0.494
FEV1%	58.6%	66.5%	70.2%	0.256
CF liver disease [10]	42.9%	36.4%	56.0%	0.381
Nasal polyps	25.0%	27.3%	40.0%	0.457
GERD	25.0%	27.3%	36.0%	0.659
Diabetes	21.4%	9.1%	24.0%	0.379
*P. aeruginosa* colonization	78.6%	72.7%	48.0%	0.048

BMI—body mass index; FEV1%—forced expiratory volume in 1 s; GERD—gastroesophageal reflux disease; and MCT—medium-chain triglycerides.

**Table 3:** Doses of fat-soluble vitamins in CF patients before the trial. Medians (1st–3rd quartiles) are shown along with the *p*-value for the Kruskal–Wallis test.

Vitamin	Liposomes (*n* = 28)	Cyclodextrins (*n* = 22)	MCT (*n* = 25)	*p*
Vitamin A, IU/d, incl. β-carotene	5000 (0–7492)	6662 (0–10,500)	5000 (2000–10,000)	0.30
Vitamin D, IU/d	3000 (2000–4100)	3000 (2000–5000)	3000 (2000–5000)	0.92
Vitamin E, mg/d	270 (135–400)	236 (135–300)	236 (100–400)	0.99
Vitamin K1, mean daily mg/d	1.43 (0.10–4.64)	1.71 (0.20–4.14)	1.63 (1.43–2.86)	0.91

MCT—medium-chain triglycerides.

**Table 4:** Vitamin A, D, E, and K status (serum) in CF patients before and after supplementation. Medians (1st–3rd quartile) are given along with means ± standard deviation. Statistical significance for the increase/decrease is given in Figure 2. Statistical significance (two-sided *p*-value) for direct comparisons of values using the Welch test is provided.

Parameter	Liposomes (*n* = 26–28)	Cyclodextrins (*n* = 18–22)	MCT (*n* = 23–25)	p_LIPvsMCT_	p_CYKvsMCT_	p_LIPvsCYK_
Start: all-trans-retinol, ng/mL	374 (294–448) 381 ± 98	338 (291–413) 376 ± 129	370 (325–422) 360 ± 107	0.281	0.509	0.869
End: all-trans-retinol, ng/mL	382 (322–499) 418 ± 131	360 (260–373) 346 ± 107	330 (292–379) 327 ± 83	0.005	0.541	0.052
Start: 25-OHD3, ng/mL	31.0 (23.0–36.0) 30.2 ± 11.3	27.0 (21.0–35.0) 26.7 ± 9.4	32.0 (22.0–35.0) 29.4 ± 11.8	0.802	0.428	0.274
End: 25-OHD3, ng/mL	31.0 (25.0–46.0) 34.1 ± 13.2	35.5 (29.0–42.0) 35.9 ± 8.7	30.0 (25.0–37.0) 30.5 ± 10.6	0.296	0.083	0.594
Start: α-tocopherol, µg/mL	*8.32 (6.28–13.11) (Tokovit)* *9.80 ± 4.33*	7.99 (6.55–10.86) 9.39 ± 4.22	8.81 (6.03–10.44) 8.57 ± 3.24	*0.329*	0.937	*0.330*
End: α-tocopherol, µg/mL	*11.52 (9.32–13.58) (Tokovit)* *11.44 ± 3.63*	11.22 (5.47–17.16) 12.4 ± 7.0	7.47 (5.24–12.21) 9.14 ± 4.48	*0.057*	0.099	*0.611*
Start: %ucOC	26.2 (11.1–64.7) 37.0 ± 30.0	48.0 (15.3–58.8) 39.7 ± 24.3	29.8 (13.3–67.3) 42.1 ± 30.1	0.559	0.785	0.740
End: %ucOC	27.0 (7.7–41.8) 30.0 ± 24.8	29.3 (11.2–64.8) 37.5 ± 26.9	28.1 (19.5–73.9) 41.4 ± 28.8	0.146	0.650	0.357

%ucOC—the percentage of undercarboxylated prothrombin; 25-OHD3—25-hydroxyvitamin D3; CYK—cyclodextrins; LIP—liposomes; and MCT—medium-chain triglycerides.

**Table 5:** The primary outcome. Changes in the concentrations of all-trans-retinol, 25-hydroxyvitamin D3 (25-OHD3), α-tocopherol, and the percentage of undercarboxylated osteocalcin (%ucOC) in the serum of PI CF patients supplemented for three months with vitamins in cyclodextrin, liposomal, or MCT forms. Liposomal vitamin E could not be investigated for technical reasons. Medians (1st–3rd quartile) are presented along with means ± standard deviation and two-sided *p* values for the comparisons using the Welch test. Studied doses of vitamin A and E were lower than that typically used by the patients before inclusion.

Parameter	Liposomes	p_LIPvsMCT_	Cyclodextrins	p_CYKvsMCT_	MCT	p_LIPvsCYK_
Δ all-trans-retinol, ng/mL	1.7 (−44.3–86.1) 26.2 ± 114.4	0.028	−22.5 (−81.2–20.6) −39.7 ± 96.3	0.787	−38.8 (−71.2–6.8) −32.6 ± 61.4	0.045
Δ 25-OHD3, ng/mL	2.0 (−1.0–1.0) 3.7 ± 10.0	0.330	9.0 (1.0–17.0) 9.1 ± 10.2	0.012	3.0 (−4.0–7.0) 1.1 ± 8.8	0.095
Δ α-tocopherol, µg/mL	*0.92 (−0.73–4.73)* *1.68 ± 3.94*	*0.206*	4.34 (0.33–6.52) 3.75 ± 4.44	0.010	−0.34 (−1.71–2.15) 0.44 ± 2.73	*0.121*
Δ %ucOC (increased in deficiency)	−2.5 (−9.9–2.6) −7.0 ± 26.3	0.367	−2.7 (−18.8–18.3) −2.2 ± 28.7	0.842	3.5 (−11.8–9.8) −0.6 ± 22.7	0.583

CYK—cyclodextrins; LIP—liposomes; and MCT—medium-chain triglycerides.

**Table 6:** The secondary outcome. Changes in the frequency of vitamin A, D, E, and K insufficiencies in PI CF patients during three-month supplementation of fat-soluble vitamins in liposomes, cyclodextrins, and MCT. In the liposomal group, standard vitamin E preparation was used for technical reasons. Frequencies at the start and end of the intervention are provided. None of the differences were statistically significant (Fisher’s exact test). Studied doses of vitamin A and E were lower than that typically used by the patients before inclusion.

Vitamin	Liposomes	Cyclodextrins	MCT
Vitamin A insufficiency	23.1% → 15.4%	22.2% → 33.3%	26.1% → 34.8%
Vitamin D insufficiency	12.0% → 8.0%	22.0% → 0%	13.0% → 13.0%
Vitamin E insufficiency	*3.8% * * → 0%*	5.6% → 22.2%	8.7% → 13.0%
Vitamin K insufficiency	57.7% → 53.8%	66.7% →66.7%	65.2% → 73.9%

MCT—medium-chain triglycerides.

## Data Availability

The data may be available at a reasonable request.
